# Comparison of the *in vitro *invasive capabilities of *Plasmodium falciparum *schizonts isolated by Percoll gradient or using magnetic based separation

**DOI:** 10.1186/1475-2875-10-96

**Published:** 2011-04-18

**Authors:** Carmenza Spadafora, Lucia Gerena, Karen M Kopydlowski

**Affiliations:** 1 Cellular Injury Department, Division of Military Casualty Research, Walter Reed Army Institute of Research, 503 Robert Grant Ave., Silver Spring, MD 20902, USA; 2 Centro de Biologia Celular y Molecular de las Enfermedades, Instituto de Investigaciones Científicas y Servicios de Alta Tecnología-AIP (INDICASAT-AIP), Ciudad del Saber, Clayton, Panamá; 3 Parasitology Department, Division of Experimental Therapeutics, Walter Reed Army Institute of Research, 503 Robert Grant Ave., Silver Spring, MD 20902, USA; 4 US Army Medical Materiel Development Activity (USAMMDA), Fort Detrick, MD 21702, USA

## Abstract

**Background:**

Percoll gradient centrifugation is often used for synchronization, enrichment, or isolation of a particular stage of *Plasmodium falciparum*. However, Percoll, a hyperosmotic agent, may have harmful effects on the parasites. Magnetic bead column (MBC) separation has been used as an alternative. This is a report of a head-to-head comparison of the *in vitro *invasive capabilities of parasites isolated by either of the two methods.

**Methods:**

The *P. falciparum *laboratory strain isolate 7G8 was grown *in vitro *using standard procedures and synchronized using 5% sorbitol. On separate days when the schizont parasitaemia was >1%, the culture was split and half was processed by Percoll gradient centrifugation and the other half by magnetic bead column separation. Both processed parasites were placed back in culture and allowed to invade new uninfected erythrocytes.

**Results:**

In 10 paired assays, the mean efficiency of invasion of 7G8 parasites treated by Percoll gradient centrifugation was 35.8% that of those treated by magnetic bead column separation (95% CI, p = 0.00067) A paired *t *test with two tails was used for these comparisons.

**Conclusions:**

In this comparison, magnetic bead column separation of 7G8 schizonts resulted in higher viability and efficiency of invasion than utilizing Percoll gradient centrifugation.

## Background

Purification of schizonts from *in vitro *cultures of *Plasmodium falciparum *is desirable for a number of reasons: to synchronize the culture and aid in the study of the stage specific proteome and transcriptome, to carry out red cell invasion assays in order to study mechanisms of merozoite invasion, to test the growth inhibitory activity of antibodies or anti-sera, and to determine the stage specific efficacy of potential anti-malarial drugs. To achieve synchronization several methods have been developed. One of the earliest reported is based on the use of a gelatin [1], which relies on the different sedimentation speeds of the infected erythrocytes dependent on the age of the parasite inside. It was reported that a better substitute (i.e., Plasmion) of the common, but discontinued Plasmagel, works equally well regardless of the strain used [2], since only membrane knobs expressing strains could be separated with Plasmagel [3]. The most common method used by researchers to synchronize their cultures is incubation with 5% sorbitol [4], which destroys the late stages of the parasite (i.e., throphozoites and schizonts). This method is very toxic to the parasites, though, and the researcher usually has to allow for the parasites to complete up to two more reproduction cycles for full recovery from the treatment [5-7]. Another way of separating the stages is through the use of a discontinuous gradient of Percoll [8] with or without sorbitol, or in combination with glucose. Percoll gradient centrifugation became the method of choice by most malaria researchers when trying to separate different stages of the parasitized erythrocytes. A method based on the magnetic properties of the late trophozoite or schizont stage of the malaria parasite was later described by Staalsoe [9], and the viability of the parasites treated this way was tested *in vivo *by Thi Trang [10]. The use of magnetic bead columns to capture malaria infected erythrocytes is possible because *Plasmodium *parasites utilize the haemoglobin of the red blood cells as a nutrient and transform the iron containing haem group into haemozoin, a pigment that remains in the food vacuole. Therefore, schizonts possess an abundance of the iron-containing haemozoin. Because the iron in haemozoin is in the Fe^3+ ^state, it has a stronger paramagnetic effect than the iron in haemoglobin, which is in the Fe^2+ ^state. This translates into a stronger magnetic attraction under a magnetic field for haemozoin than for haemoglobin. Because not all strains of *P. falciparum *seem to withstand well the Percoll treatment [11], the *in vitro *retained capability of invasion of new uninfected erythrocytes of the 7G8 strain parasites after purification either by Percoll gradient centrifugation or magnetic bead column separation was compared.

## Methods

### Parasites and culture

*Plasmodium falciparum *chloroquine-resistant and pyrimethamine-resistant strain 7G8 [12] was used throughout. The parasites were grown as per the method described by Haynes *et al *[13] using O+ red blood cells at 2-4% haematocrit in RPMI 1640 containing 25 mM Hepes, 2% glucose, 0.2% sodium bicarbonate, (culture media, CM) plus 10% O+ plasma or serum. Semi-synchronized cultures with 1-10% parasitaemia were used to isolate RBCs infected with late stages of the parasite. Giemsa-stained smears of the culture and flow cytometry by Hoechst 33342 staining of the purified parasites were prepared as needed to evaluate parasitaemia.

### Percoll gradient and magnetic column separation

Cultures with parasitaemia of 4-20% were divided into two equal portions. One half was used for Percoll gradient centrifugation and the other for magnetic bead column separation. The Percoll gradient consisted of three layers of Percoll in RPMI (from top to bottom: 40% (4 ml), 70% (6 ml) and 90% (8 ml) Percoll) placed in 30 ml OAK RIDGE polycarbonate screw cap centrifuge tubes. The culture was centrifuged at 3000 rpm for 5 min at 4°C, the pellet resuspended in RPMI-1640 (1:1 vol/vol), and carefully placed on top of the gradient. The tubes were centrifuged at 10,000 rpm for 20 min at 20°C. The cells at the 40-70% interface, containing mostly schizonts, were collected, washed twice in RPMI-1640, resuspended in culture medium, counted in a haemocytometer and used as inoculum for the overnight cultures. For magnetic column separation, we used MACS Separation Columns "LS" (Miltenyi Biotec, Germany). Pre-equilibration, washings, and elution were all carried out at RT with CM. Upon applying a magnetic field, three 8 ml loads of culture were added to the column and the flow-through, which usually contained uninfected erythrocytes, ring-infected erythrocytes, and young trophozoites, was collected and used to reload the column or was placed back in culture. After one wash with 8 ml of CM (since then we have optimized the purity obtained with the procedure by washing first with one ml followed by 5 ml of CM), the column was removed from the magnetic field and its contents eluted with 3 ml of CM. The eluted parasitized RBCs were centrifuged at 1,500 rpm for 5 min at RT, the supernatant discarded, and the pellet resuspended in 150-250 μl of CM plus 10% human serum (CMS), depending on the concentration desired. The number of parasites captured was calculated by haemocytometer determination, as with the Percoll treatment.

### Invasion assays

Schizont-infected erythrocytes from both magnetic beads column or Percoll gradient separation were incubated for 16-22 hrs at a haematocrit of 1-2% in CMS, in duplicate wells. Final schizont-infected parasitaemia ranged from 1 to 10%. For light microscopy readings, Giemsa-stained thin smears were prepared from each well and the number of ring-infected erythrocytes in 1,000 erythrocytes was counted. The microscopist was always blinded to the experimental group designation of each slide. For the flow cytometry readings, infected red blood cells were stained with Hoechst 33342 and read with a UV laser in a BD LSRII equipment following procedures as described by Spadafora *et al *[14]. Each experiment was set up in duplicate or triplicate wells, and invasion rates were averaged between all the wells.

### Statistical analysis

The percentage of efficiency of invasion compared to the MCB method is the result of the following formula:

A paired t-test analysis was performed, where each sample from Percoll and from MBC done on the same day was compared to each other for their invasion percent into new red blood cells. Only experiments with purity of isolation above 65% were considered for the calculations.

## Results and Discussion

Table 1 shows the results of the experiments. In 10 paired assays, the mean percent invasion of intact red cells after overnight culture was 5.3% for magnetic bead column separation and 1.9% for Percoll gradient centrifugation, accounting for a 39.5% efficiency of invasion by the parasites treated with the Percoll method compared to the magnetic beads one (95% CI, P = 0.00067).

**Table 1:** Relationship between *P. falciparum *capacity of invasion after two different separation treatments

Experiment	Initial Parasitaemia	Replicates	Percoll Invasion	MB invasion
*1	10%	2	0.8%	8.2%
*2	4%	2	0.6%	2.0%
*3	2%	3	1.3%	2.4%
*4	2%	3	1.9%	3.8%
5	2%	2	2.0%	6.7%
6	5%	2	4.9%	7.3%
7	1%	2	0.8%	4.4%
8	2%	3	2.8%	11.5%
9	2%	3	2.0%	4.1%
10	2%	3	1.7%	2.7%
Average:			1.9%	5.3%

Parasites underwent separation by use of a Percoll gradient (Percoll invasion column) or passage through a magnetic beads column (MB invasion column). The collected schizonts were then placed in culture with new red blood cells to allow for a new round of infection by the parasite. The parasitaemia was calculated by flow cytometry except for experiments marked with "*" which were determined by light microscopy.

It is evident that schizonts purified by magnetic bead column separation were more viable than those purified by Percoll gradient centrifugation. The reasons for this are not clear. However, there are several possible explanations. It hs been reported that permeabilization of the host erythrocyte membrane appears s early as 6 h after parasite invasion and increases gradually with parasite maturation [15]. Since the collection of parasites for next day invasion need collection of schizont forms, the erythrocyte is at its peak of permeability. This suggests that molecules could diffuse in and out of the schizont-infected erythrocyte with relative ease, perhaps leading to increased susceptibility to toxic substances that could be present in Percoll. Another possible explanation for the decreased viability after Percoll centrifugation is the leakage of ions across the membrane due to the hyper osmotic effect of Percoll.

Bates *et al *[16] reported that a group of laboratory established strains of *P. falciparum *treated by two methods of isolation, MCB and a centrifugation gradient obtained with Percoll to which sorbitol has been added, did not show any difference in their invasion capabilities. However, they reported that when they performed the same analysis with field isolates their results were' similar to those we show here, with the MCB treated ones invading at higher rates. It seemed to them that the adaptation to the laboratory conditions was the major factor determining if the parasites would be affected or not by the Percoll/sorbitol treatment. In view of the present experiments, these results are better explained by the difference in susceptibility to external chemicals and solvents inherent to different strains. Reed *et al *[11] reported that the laboratory-adapted strain W2*mef *could not be isolated using the Percoll method, which would also point out to different strains behaving differently to the Percoll treatment, for reasons that are still unknown, and that could include different membrane porosity and osmotic pressure sensitivity. In the case of 7G8, the isolation is possible but the invasion efficiency is reduced, something that could be avoided using the magnetic beads based technique.

In addition to increased viability, magnetic bead column separation offers the advantage that the parasitized red cells remain in an iso-osmotic environment. This means that the red cells can be introduced back into the culture media without concern for abrupt changes in the osmolarity, which could affect some strains more than others.

## Conclusions

Magnetic column enrichment of schizont and late stage trophozoite-infected red cells is a very viable alternative to Percoll gradient centrifugation especially for the purpose of preparing red cell invasion assays with the 7G8 strain. This technique can be recommended for several reasons: the procedure itself has the advantage of not being invasive on the parasites since it involves no contact with any foreign chemical reagent to the culture media, it requires no expensive centrifuges, and 7G8, as well as some other strains seem to be affected by the use of Percoll gradient centrifugation.

## Competing interests

The authors declare that they have no competing interests.

## Authors' contributions

CS suggested the idea, developed the protocol for the magnetic beads separation, maintained *in vitro *culture, carried out most parts of the laboratory experiments, prepared and analyzed the data obtained, drafted the paper, and finalized the manuscript for submission with contribution from authors. KMK was involved in the experimental design, participated in the coordination of the project under which this study was conducted, assisted in the management of culture, helped supervise, and contributed to the preparation of the manuscript. LG contributed with maintaining parasite culture and assisted with interpretation of smears. All authors read and approved the final manuscript.
